# Supplementary material for: Screening accuracy of a 14-day smartphone ambulatory assessment of depression symptoms and mood dynamics in a general population sample: Comparison with the PHQ-9 depression screening
Source: PLoS One. 2021 Jan 6;16(1):e0244955. doi: 10.1371/journal.pone.0244955 (PMC7787464; doi:10.1371/journal.pone.0244955)
Supplement: S1 Fig — Assessment time = Exact time point of assessment; Mood rating = Momentary assessed mood score between 0 (negative mood) and 5 (positive mood). (PDF) [file pone.0244955.s001.pdf]

S1 Fig. Individual mood trajectories over the 14-day assessment period (N = 113).

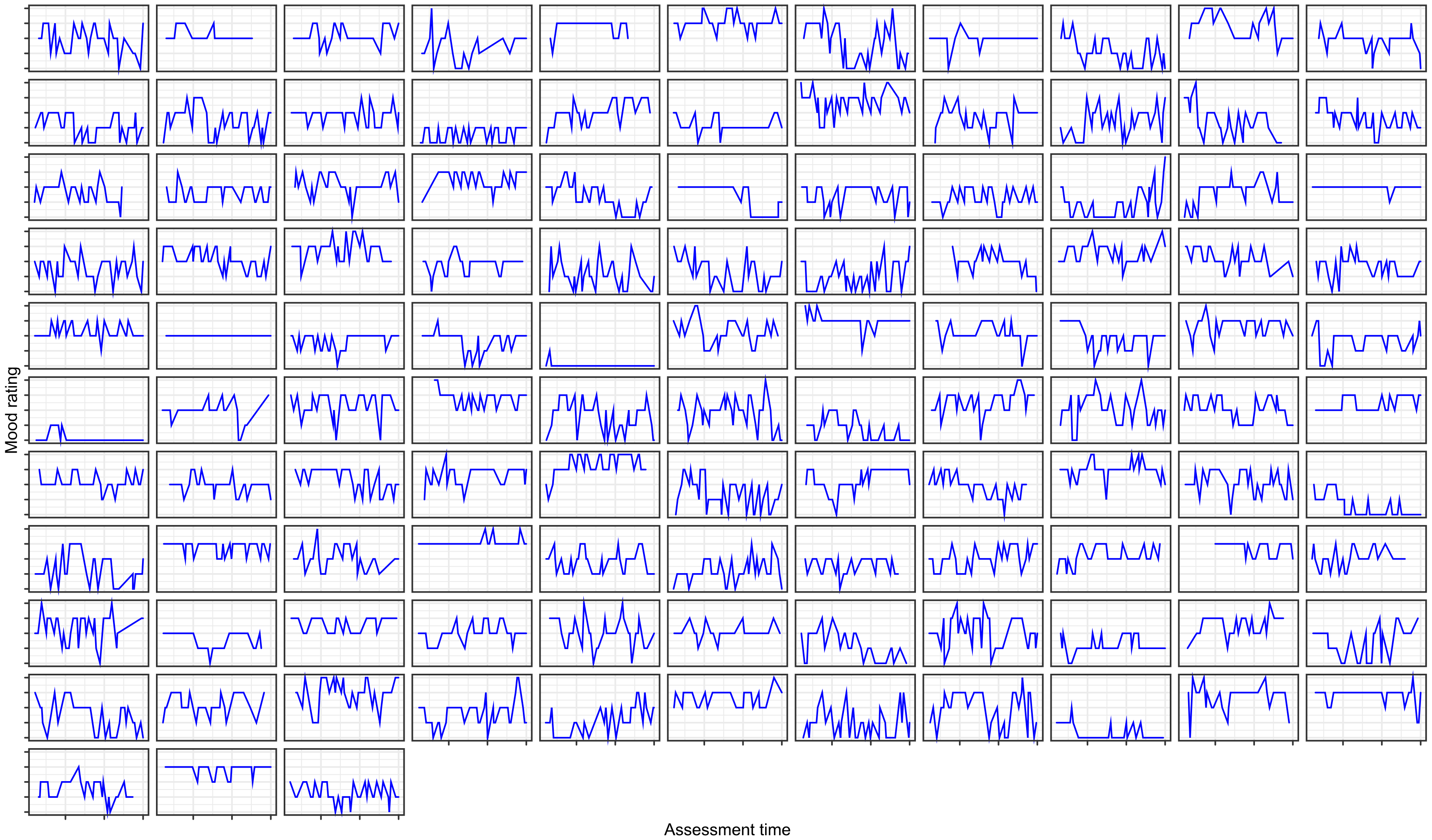

Assessment time = Exact time point of assessment; Mood rating = Momentary assessed mood score between 0 (negative mood) and 5 (positive mood)
