# Supplementary figures and images for: Screening accuracy of a 14-day smartphone ambulatory assessment of depression symptoms and mood dynamics in a general population sample: Comparison with the PHQ-9 depression screening
Source: PLoS One. 2021 Jan 6;16(1):e0244955. doi: 10.1371/journal.pone.0244955 (PMC7787464; doi:10.1371/journal.pone.0244955)

**S2 Fig. Mood RMSSD for levels of depression severity (N = 113).**

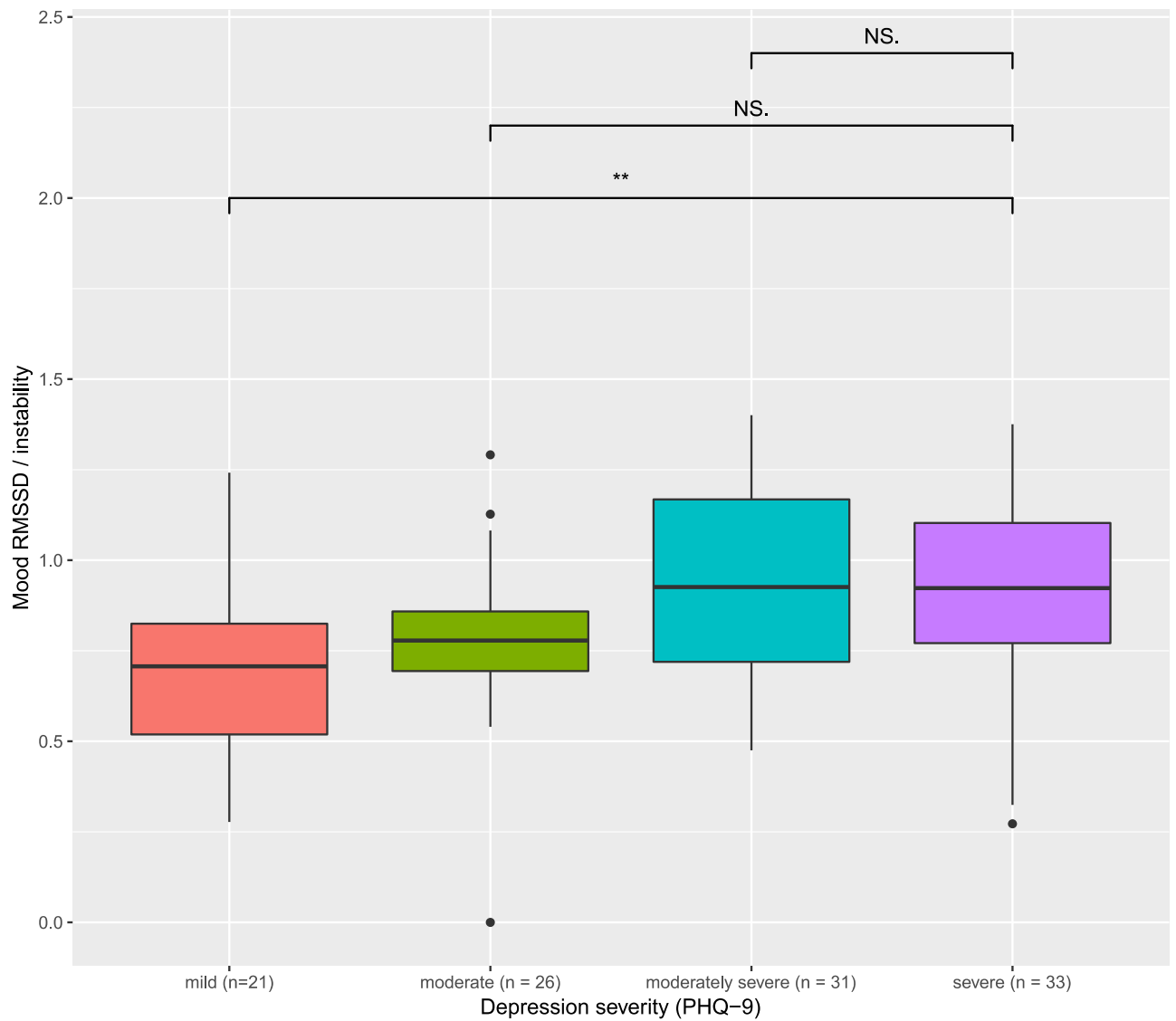

NS  $p \geq .05$ , \*\*  $p < .01$ .

Supplement: S2 Fig — NS p ≥ .05, ** p < .01. (PDF) [file pone.0244955.s002.pdf]

**S3 Fig. Mood SD for levels of depression severity (N = 113).**

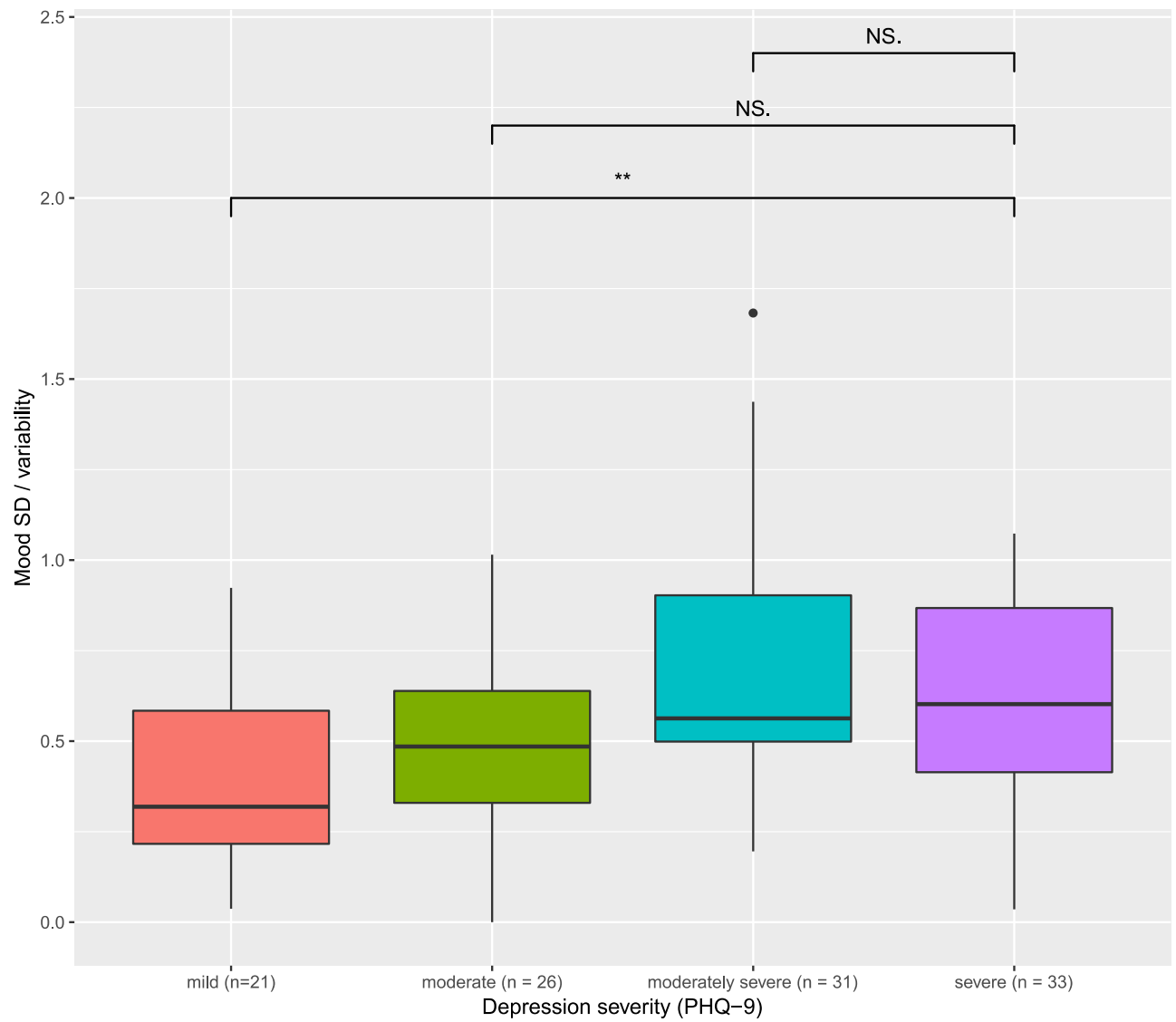

NS  $p \geq .05$ , \*\*  $p < .01$ .

Supplement: S3 Fig — NS p ≥ .05, ** p < .01. (PDF) [file pone.0244955.s003.pdf]

**S4 Fig. Mood average for levels of depression severity (N = 113).**

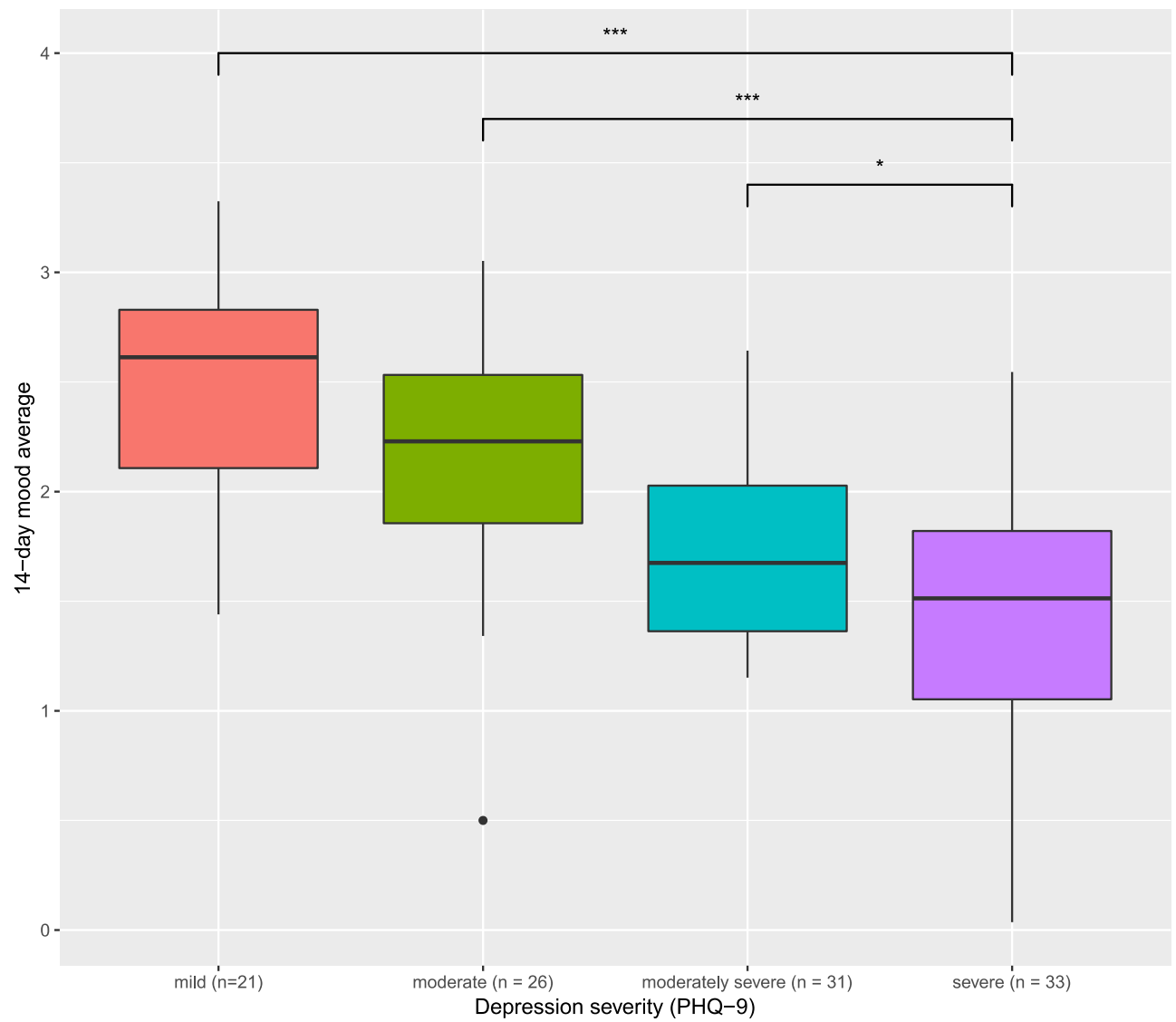

\*  $p < .05$ , \*\*\*  $p < .001$ .

Supplement: S4 Fig — * p < .05, *** p < .001. (PDF) [file pone.0244955.s004.pdf]

**S5 Fig. Mood inertia for levels of depression severity (N = 113).**

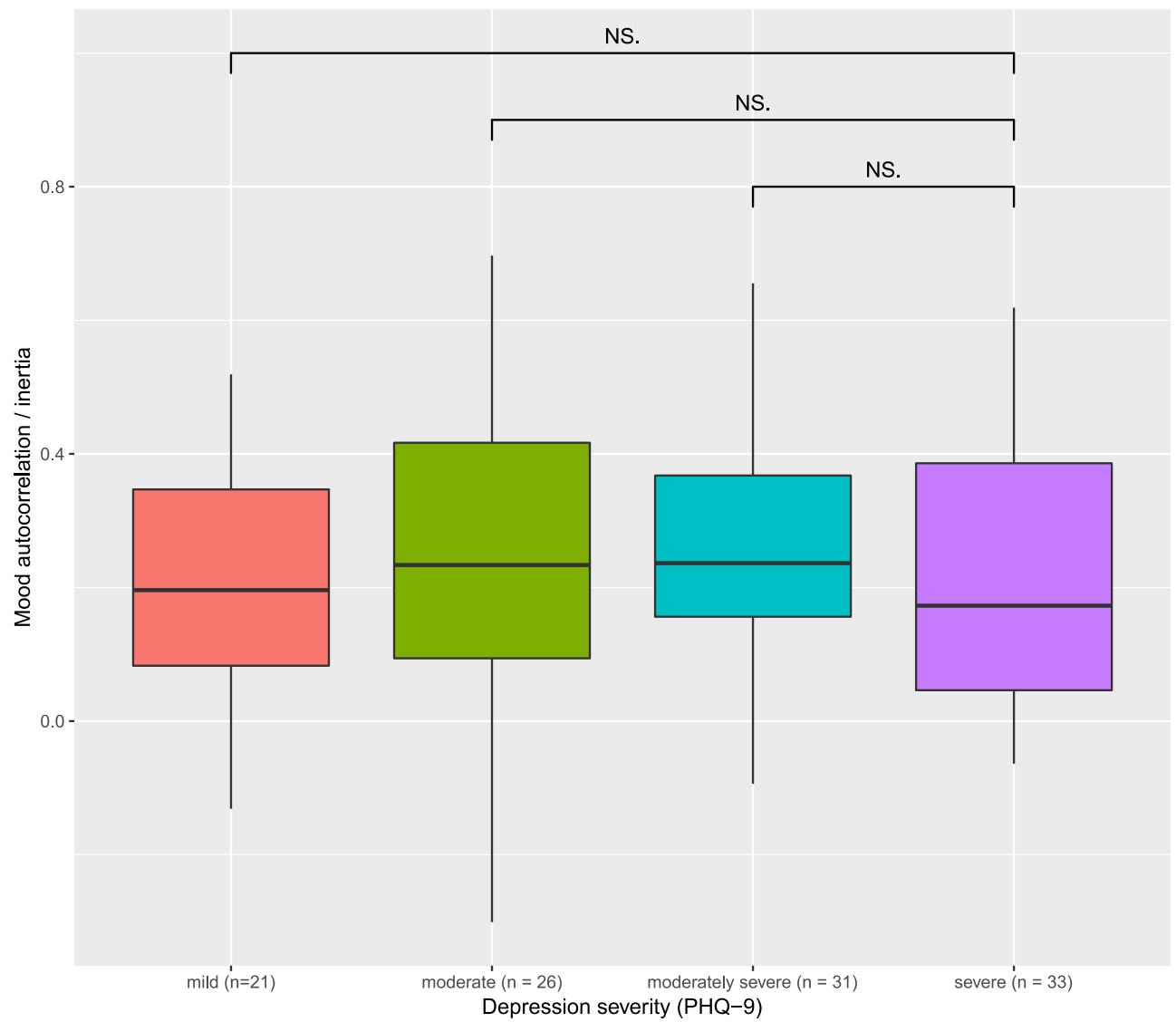

NS  $p \geq .05$ .

Supplement: S5 Fig — NS p ≥ .05. (PDF) [file pone.0244955.s005.pdf]
