# Supplementary material for: Screening accuracy of a 14-day smartphone ambulatory assessment of depression symptoms and mood dynamics in a general population sample: Comparison with the PHQ-9 depression screening
Source: PLoS One. 2021 Jan 6;16(1):e0244955. doi: 10.1371/journal.pone.0244955 (PMC7787464; doi:10.1371/journal.pone.0244955)
Supplement: S1 Table — (PDF) [file pone.0244955.s006.pdf]

S6 Table. List of the 45 questions used for Ambulatory Assessment.

Question #	**Symptome der Depression**
1	Fühlst Du Dich gerade niedergeschlagen oder traurig?
2	Fühlst Du Dich gerade hoffnungslos?
3	Hast Du gerade das Gefühl, zu nichts mehr Lust zu haben?
4	Hast Du gerade weniger oder gar keine Freude an Aktivitäten, die Dir sonst Spaß machen?
5	Hast Du gerade weniger oder gar keine Energie?
6	Ermüden Dich gerade selbst alltägliche Aufgaben?
7	Fällt es Dir gerade schwer, Entscheidungen zu treffen?
8	Fällt es Dir gerade schwer, Dich zu konzentrieren?
9	Traust Du Dir gerade weniger oder gar nichts mehr zu?
10	Fühlst Du dich gerade Deinen Aufgaben gewachsen?
11	Machst Du Dir gerade Selbstvorwürfe?
12	Glaubst Du gerade, dass Du weniger wert bist als andere Menschen?
13	Glaubst Du gerade, dass es Dir in der Zukunft gut gehen wird?
14	Schaust Du gerade hoffnungsvoll in die Zukunft?
15	Denkst Du häufiger an den Tod?
16	Hast Du in der letzten Nacht schlecht geschlafen?
17	Hast Du heute weniger oder gar keinen Appetit?
	**Somatisches Syndrom**
18	Ist Deine Stimmung in letzter Zeit morgens deutlich schlechter als am Rest des Tages?
19	Verlierst Du in letzter Zeit ungewollt an Körpergewicht?
20	Sprichst oder bewegst Du dich deutlich langsamer als sonst?
21	Fühlst Du dich innerlich unruhig, überdreht oder ruhelos?
22	Hast Du in letzter Zeit deutlich weniger Interesse am Sex als sonst?
	**Weitere mit der Depression assoziierte Symptome und Risikofaktoren**
23	Musstest Du in letzter Zeit häufig weinen?
24	Denkst Du in letzter Zeit häufig über die gleichen Themen oder Probleme nach, ohne zu einer Lösung zu kommen?
25	Hast Du das Gefühl, weder Freude noch Trauer zu empfinden?
	**Screening Fragen zu anderen Störungsbildern**
26	Hattest Du schon einmal eine plötzliche Angstattacke?
27	Machst Du Dir häufig Sorgen um Deine körperliche Gesundheit?
28	Hast Du häufig Angst davor, in Gegenwart anderer Menschen zu sprechen?
29	Hast Du häufig Angst davor, dich auf öffentlichen Plätzen oder in öffentlichen Verkehrsmitteln aufzuhalten?
30	Leidest Du häufig unter Erinnerungen an ein belastendes Ereignis in der Vergangenheit?
31	Hast Du häufig Angst vor einem bestimmten Gegenstand, einem Tier oder einer bestimmten Situation?

32	Belasten Dich häufig Gedanken oder Handlungen, die eigentlich unsinnig sind, sich aber oft ungewollt wiederholen?
33	Warst Du jemals über mehrere Tage hinweg ungewöhnlich glücklich, aktiv, überdreht oder reizbar?
34	Machst Du Dir häufig Sorgen um Dein Aussehen, Dein Körpergewicht oder Deine Figur?
35	Konsumierst Du häufig größere Mengen an Alkohol, Drogen oder Medikamenten?
	**Sport**
36	Machst Du in letzter Zeit regelmäßig Sport?
	**Soziale Unterstützung**
37	Wenn Du traurig bist, gibt es Menschen, die Dich aufmuntern?
38	Hast Du Menschen, auf die Du dich verlassen kannst?
	**Wohlbefinden**
39	Bist Du froh und guter Laune?
40	Fühlst Du dich ruhig und entspannt?
41	Fühlst Du dich energisch und aktiv?
42	Hast Du dich beim Aufwachen frisch und ausgeruht gefühlt?
43	Ist Dein Alltag voller Dinge, die Dich interessieren?
	**Beeinträchtigung**
44	Fühlst Du dich bei familiären und häuslichen Verpflichtungen beeinträchtigt? (z.B. Hausarbeit, Kindererziehung)
45	Fühlst Du dich bei der Bewältigung von Arbeit und Beruf beeinträchtigt? (z.B. Leistungsfähigkeit, Anforderungen erfüllen)
