# Supplementary material for: Screening accuracy of a 14-day smartphone ambulatory assessment of depression symptoms and mood dynamics in a general population sample: Comparison with the PHQ-9 depression screening
Source: PLoS One. 2021 Jan 6;16(1):e0244955. doi: 10.1371/journal.pone.0244955 (PMC7787464; doi:10.1371/journal.pone.0244955)
Supplement: S2 Table — (PDF) [file pone.0244955.s007.pdf]

S7 Table. Order of the AA question blocks.

Day	Block	Question #			Priority
1	morning	2	5	10	obligatory
	midday	3	17	21	obligatory
	evening	1	12	7	obligatory
2	morning	9	16	23	obligatory
	midday	15	29	20	obligatory
	evening	7	17	35	obligatory
3	morning	12	8	16	optional
	midday	6	31	33	obligatory
	evening	3	4	28	obligatory
4	morning	1	10	16	optional
	midday	17	24	13	obligatory
	evening	5	21	32	obligatory
5	morning	8	16	18	obligatory
	midday	1	9	15	obligatory
	evening	11	27	14	obligatory
6	morning	4	16	41	optional
	midday	10	20	13	obligatory
	evening	5	38	14	optional
7	morning	16	19	36	obligatory
	midday	9	7	13	optional
	evening	30	34	22	obligatory
8	morning	11	17	26	obligatory
	midday	4	8	21	obligatory
	evening	1	3	17	optional
9	morning	2	15	14	obligatory
	midday	5	39	37	optional
	evening	11	17	40	optional
10	morning	16	20	25	obligatory
	midday	2	9	8	optional
	evening	17	44	13	optional
11	morning	6	12	15	obligatory
	midday	1	11	45	optional
	evening	6	10	14	optional
12	morning	3	7	16	optional
	midday	2	10	43	optional
	evening	3	9	8	optional
13	morning	4	16	17	optional
	midday	11	17	14	optional
	evening	5	7	13	optional
14	morning	6	12	16	optional
	midday	4	6	42	optional
	evening	2	12	17	optional
