# Supplementary material for: Screening accuracy of a 14-day smartphone ambulatory assessment of depression symptoms and mood dynamics in a general population sample: Comparison with the PHQ-9 depression screening
Source: PLoS One. 2021 Jan 6;16(1):e0244955. doi: 10.1371/journal.pone.0244955 (PMC7787464; doi:10.1371/journal.pone.0244955)
Supplement: Attachment — Submitted filename: PONE-D-19-24772 Review.pdf [file pone.0244955.s008.pdf]

PONE-19-24772

GENERAL COMMENTS

This paper reports on a diagnostic accuracy study which evaluates the ability of self-reported ambulatory assessment of depressive symptoms and mood monitoring to correctly classify participants as experiencing Major Depressive Disorder, using the PHQ-9 as the gold standard.

This paper is timely as the use of ambulatory assessment and momentary monitoring of mood symptoms is gaining in use ahead of the research evidence which should underpin it.

The paper is fairly well written but it is long and could be shortened to improve clarity. The main concern I have is whether there might be two papers presented as one here. Simply put, one is whether MOODPATH diagnosis agrees with a PHQ-9 diagnosis and the other is whether mood monitoring agrees with the PHQ-9 scores? It took me a number of times reading through the manuscript to understand exactly what was being presented and whether they actually belonged together? I just wonder if you are trying to achieve too much for one paper and clarity could be improved if you dealt with them separately? Another concern is the large number of models presented and the lack of a pre-published protocol. This needs to be addressed.

SPECIFIC COMMENTS

I have arranged my feedback using the STAR D checklist

Section & Topic	No	Item	Reported on page #
**TITLE OR ABSTRACT**			
	**1**	The title could be altered to clearly identify this paper as a study of diagnostic accuracy. E.<br><br>The diagnostic accuracy of 14-day smartphone ambulatory assessment of depression symptoms and mood dynamics comparison with the PHQ-9 depression screening in a population sample.	1 of 36
**ABSTRACT**			
	**2**	The abstract overstates the background literature and could benefit from a more objective stance. Detection of cases in primary care is a complex issue. The seminal study by Thompson et al published in the Lancet in the early 2000 showed that the majority of the ‘missed cases’ of depression were at the milder end and close to diagnostic thresholds. The lack of a clear diagnostic test for depression/clear gold standard makes working out who is right and who is wrong a difficult endeavor. There is a tendency in the literature to assume that the primary care physician is ‘wrong’. I caution the authors from falling into this trap. Reconsider the use of “but cases of depression are often not detected by primary care providers”<br><br>What evidence do you have for the statements:<br>“there is a high demand for **low-threshold** but clinically sound approaches to depression detection’ ?<br>and<br>“Recent studies show **a great willingness among** users of mobile health apps..”	1
**INTRODUCTION**			
	**3**	Scientific and clinical background could be edited to be more balanced about the state of the evidence. There is a weighting towards assuming that the approach will be beneficial (see examples below) and only a few lines (170-172) (100-107) about the potential for harm.<br>The introduction should also include more explicitly the intended use and clinical role of the approach, being clear about the setting/population they are designing the test for.<br><br>Examples in the Introduction that are overstating the evidence:<br>In Line 94: references line 734-736 ref 19 Rubanovich to make the statement that mApps **are widely used**. When I checked this reference I found that Rubanovich and colleagues recruited for one year	

		(2015-2016) using a variety of sources (health care orgs, web ads, news stories, public transport Ads, and word of mouth, Google play store, research registries, social media, fliers and other sources. They also paid people $USD20 for completing the surveys. Despite this they report only 177 participants. The authors themselves make the statement that 300 million people worldwide experience depression. One could expect that with the huge target population and the very comprehensive recruitment strategies that Rubanovich employed they would have attracted many more participants than 177 **if mApps are widely used?** Unfortunately, Rubanovich does not indicate anywhere in their paper that I could see how many clicks or the possible audience they were 'seen by' to determine their true response rate. There is a growing tendency within the mHealth research field to overstate the use of m technologies, and it will be much more helpful for the research community if a more objective stance is taken. The potential of mhealth to make an impact is certainly there, but we are far from that reality at present and we need to build the evidence base to understand why the uptake is studies such as Rubanovich is so low.<br><br>Line 95 quotes Torous paper about smartphone ownership to support the statement that there is a high willingness to complete smartphone symptom screenings. When I checked this reference I found that 50% of 100 smart phone owners attending a psychiatric outpatient clinic of a large teaching hospital expressed interest in using smart phone symptom tracking. They also state that only 10% of patients took part in the survey. I have not checked all the references, but the tendency to overstate the findings detracts from the importance of the current paper. I suggest that the authors more accurately report the state of the evidence.<br><br>Line119: it would be helpful if the strengths and weaknesses of this approach could be clearly articulated here and referenced appropriately.	
	**4**	**Study objectives and hypotheses**<br>The two aims are clearly stated and the use of 14-day monitoring compared with the retrospective 14 day PHQ is a strength.	Line 177
**METHODS**		*Some of the details here should be in the results e.g. N=200, lines 202-214 are results. Reorganise the paper to be in line with the STAR D guidance. 202-214 move to results*	
Study design	**5**	It appears that this is a retrospective design – this is worth stating and including in the abstract.<br>Can you explain why you decided to use MOODPATH users for this study<br>Can you confirm whether any of the authors have any connection to Moodpath or Aurora	
		Please organise the participants section in line with the STARD flow as below.	
Participants	**6**	Eligibility criteria – users of Moodpath App and agree to consent – can this be stated clearly?	
	**7**	On what basis potentially eligible participants were identified<br>This is well described.	
	**8**	Where and when potentially eligible participants were identified (setting, location and dates)	
	**9**	Whether participants formed a consecutive, random or convenience series<br>Please make it clear that this is a convenience sample.	
Test methods	**10a**	Index test, in sufficient detail to allow replication<br><br>You have two index tests MOODPATH and the mood dynamics – or is the mood dynamics a part of the MOODPATH assessment. In Table 1 are they Yes, No answers? Can you include this in the table?<br><br>Line 224 – provide reference for usability testing results<br>Line 226 – provide reference for Aurora<br>Line 229 – provide the 45 questions, even if as a supporting information document – readers need enough information to replicate your study.<br>Line 240 – can you explain how the 3 questions are selected? Really non-randomly? Do people cover all 45 questions over the 14 days in equal amounts? I found this difficult to follow. Can this be explained more clearly? Line 256-258 explains this more clearly, but this whole section could benefit from clarity and perhaps a diagram to allow replication.<br>Line 265 – explain how you ascertained 'clinical significance' for each item<br>Please justify and provide a reference to support how MOODPATH came up with all the rules they have for allocating a score?	

		Line 280 – provide justification and a reference for this approach	
	**10b**	Reference standard, in sufficient detail to allow replication<br>Line 285-296 –The PHQ-9 is a well validated scale but I found this section a little confusing.<br>The original paper is the Spitzer, JAMA 1999 paper which outlines the process for the original algorithm scoring for Major Depressive Disorder – which I noted to related to DSM IV criteria rather than DSM5?.<br>My reading of the Ref no 69 &70, is that that they provide support for using the cut-off point of >=10; but they do not appear to be an appropriate reference for using the Algorithm scoring. Can you revise this section and provide details as to whether you used the PHQ 9 as shown in the Appendix of Kroenke article Ref 32? Did you administer the section about *"… checking off how difficult these problems made it to do your work, take care of things at home etc"* This was not clear to me. How did you apply the algorithm? Please include the exact question list used as the PHQ 9 and the rating scales so replication is possible.	
	**11**	Rationale for choosing the reference standard (if alternatives exist)<br>This is explained, but you could provide a little more commentary on the reasons and perhaps the alternatives. I was also confused as to why, considering you use Ref 69&70, that you did not decide to use the cut-off of 10?	
	**12a**	Definition of and rationale for test positivity cut-offs or result categories<br>of the index test, distinguishing pre-specified from exploratory<br><br>Can you explain what you had pre-specified and what was exploratory? I find lines 300-315 hard to follow. Please make is very clear which PHQ9 scoring method you have used and which cut-off you are using.<br>Line 305: was the cut-off of 5 symptoms pre-specified? If not, please explain whether you did pre-specify a sample size and potential cut-offs that might be clinically meaningful. Explain how you decided these.<br>I became confused when I read Line 560 "The ICD-10 cut off score of 5 symptoms was used…."<br>Can you clarify?<br><br>Line 308- how did you choose the MOODPATH cut off and can state more clearly what it is?	
	**12b**	Definition of and rationale for test positivity cut-offs or result categories of the reference standard, distinguishing pre-specified from exploratory<br><br>Can you clearly state the definition and rationale for any pre-specified PHQ-9 Cut-offs you planned to use?	
	**13a**	Were clinical information and reference standard results available to the readers of the MOODPATH results?	
	**13b**	Were clinical information and index test results were available<br>to the assessors of the PHQ-9 results?	
		Did you write a separate detailed analysis plan before the analysis was conducted? Please provide this as a supplementary file.	
Analysis	**14**	Methods for estimating or comparing measures of diagnostic accuracy – these are explained.<br><br>Line 340: Can you justify the decision to calculate 14 separate regression models? How did you account for the use of multiple models? Ensure you discuss this adequately in the discussion.	
	**15**	Explain how indeterminate MOODPATH and PHQ9 results were handled<br>It would help to include a sub-heading about this and about missing data so the reader can easily find this detail.	
	**16**	Explain how missing data on the index test and reference standard were handled	
	**17**	Any analyses of variability in diagnostic accuracy, distinguishing pre-specified from exploratory<br><br>Line 303: the use of the term "ICD-based MOODPATH screening criterion of 2 core + 3 associated symptoms" is cumbersome and I found this section a little confusing. It could be written more clearly so the reader is very clear about what you are comparing with what and just how many analyses are	

		being conducted. The use of sub-headings, tables etc should be considered to increase the clarity of this section. Be consistent with terminology and consider a table of definitions to help the reader.	
	**18**	Intended sample size and how it was determined<br>- Please include details and justify your decision	
**RESULTS**			
Participants	**19**	Please include a diagram that shows the flow of participants<br><br>Include information about the number of people approached.<br>Account for all participants from the 200 to the study sample and through the data waves<br>Provide information on the MOODPATH participants – is the 113 used here representative?<br>Please move the results about the MOODPATH participants to results:<br>*In particular move lines 202-214 to results.*	
	**20**	Baseline demographic and clinical characteristics of participants<br>- Please include	
	**21a**	Distribution of severity of disease in those with the target condition<br>- Please include baseline data	
	**21b**	Distribution of alternative diagnoses in those without the target condition<br>- Please provide any details that you have about these, especially noting that the use of PHQ-9 pre-supposes that there is no physical reason for the symptoms reports.	
	**22**	Time interval and any clinical interventions between index test and reference standard<br>- Please provide details	
		This paper is presenting the comparison of MOODPATH to PHQ9 and MOOD tracking.<br>Would it be preferable to separate out these two into separate papers?	
Test results	**23**	Cross tabulation of the index test results (or their distribution)<br>by the results of the reference standard<br>- This appears in Table 2 at line 994. Please label Table 2 more clearly so it is in line with STAR D guidance.	
	**24**	Estimates of diagnostic accuracy and their precision (such as 95% confidence intervals)<br>This is included as Table 4. Please include diagnostic accuracy in the title to assist the reader to find this information easily, in line with STAR D recommendations	
	**25**	Any adverse events from performing the index test or the reference standard<br>- Please include information about how you assessed this.<br>- Include any information you have about about drop-outs	
**DISCUSSION**		*The discussion would benefit from being shortened considerably.*	
	**26**	Study limitations<br><br>There should be a section on the conundrum faced in mental health – that there is no objective gold standard, and even the best is subject to bias. This complicates all such studies.<br><br>MOODPATH – has this been developed using a rigorous process? Comment on the strengths and weaknesses of this assessment tool. Is there more work to be done?<br><br>Please comment on the study sample and how generalisble the findings are.<br><br>Comment on regression to the mean.<br>Please comment on the multiple regression models and the problems with multiple testing – how has this influenced your findings?	
	**27**	Implications for practice, including the intended use and clinical role of the index test<br>This needs to be addressed, and should be clear in the introduction also.<br>What are you wanting this tool to be used for? Please include a section on this. This will influence the interpretation of the findings and guide the future work that needs to be done.	
**OTHER INFORMATION**		*Please address these items below.*	
	**28**	Registration number and name of registry<br>- Please provide	
	**29**	Where the full study protocol can be accessed	

CC BY SA

		- I could not find any information about the study protocol	
	**30**	Sources of funding and other support; role of funders<br>Can you confirm whether any of the authors have any connection to Moodpath or Aurora?	

Information about the STAR-D checklist that might be of use from the EQUATOR website:

Explanation

A **diagnostic accuracy study** evaluates the ability of one or more medical tests to correctly classify study participants as having a **target condition.** This can be a disease, a disease stage, response or benefit from therapy, or an event or condition in the future. A medical test can be an imaging procedure, a laboratory test, elements from history and physical examination, a combination of these, or any other method for collecting information about the current health status of a patient.

The test whose accuracy is evaluated is called **index test.** A study can evaluate the accuracy of one or more index tests. Evaluating the ability of a medical test to correctly classify patients is typically done by comparing the distribution of the index test results with those of the **reference standard**. The reference standard is the best available method for establishing the presence or absence of the target condition. An accuracy study can rely on one or more reference standards.

If test results are categorized as either positive or negative, the cross tabulation of the index test results against those of the reference standard can be used to estimate the **sensitivity** of the index test (the proportion of participants *with* the target condition who have a positive index test), and its **specificity** (the proportion *without* the target condition who have a negative index test). From this cross tabulation (sometimes referred to as the contingency or "2x2" table), several other accuracy statistics can be estimated, such as the positive and negative **predictive values** of the test. Confidence intervals around estimates of accuracy can then be calculated to quantify the statistical **precision** of the measurements.

If the index test results can take more than two values, categorization of test results as positive or negative requires a **test positivity cut-off**. When multiple such cut-offs can be defined, authors can report a receiver operating characteristic (ROC) curve which graphically represents the combination of sensitivity and specificity for each possible test positivity cut-off. The **area under the ROC curve** informs in a single numerical value about the overall diagnostic accuracy of the index test.

The **intended use** of a medical test can be diagnosis, screening, staging, monitoring, surveillance, prediction or prognosis. The **clinical role** of a test explains its position relative to existing tests in the clinical pathway. A replacement test, for example, replaces an existing test. A triage test is used before an existing test; an add-on test is used after an existing test.

Besides diagnostic accuracy, several other outcomes and statistics may be relevant in the evaluation of medical tests. Medical tests can also be used to classify patients for purposes other than diagnosis, such as staging or prognosis. The STARD list was not explicitly developed for these other outcomes, statistics, and study types, although most STARD items would still apply.

DEVELOPMENT

This STARD list was released in 2015. The 30 items were identified by an international expert group of methodologists, researchers, and editors. The guiding principle in the development of STARD was to select items that, when reported, would help readers to judge the potential for bias in the study, to appraise the applicability of the study findings and the validity of conclusions and recommendations. The list represents an update of the first version, which was published in 2003.

More information can be found on http://www.equator-network.org/reporting-guidelines/stard.
