# Supplementary material for: Screening accuracy of a 14-day smartphone ambulatory assessment of depression symptoms and mood dynamics in a general population sample: Comparison with the PHQ-9 depression screening
Source: PLoS One. 2021 Jan 6;16(1):e0244955. doi: 10.1371/journal.pone.0244955 (PMC7787464; doi:10.1371/journal.pone.0244955)
Supplement: Attachment — Submitted filename: Response_to_reviewers.docx [file pone.0244955.s009.docx]

**Response to editor**

**1. When submitting your revision, we need you to address these additional requirements.
Please ensure that your manuscript meets PLOS ONE's style requirements, including those for file naming. The PLOS ONE style templates can be found at**
[**http://www.journals.plos.org/plosone/s/file?id=wjVg/PLOSOne_formatting_sample_main_body.pdf**](http://www.journals.plos.org/plosone/s/file?id=wjVg/PLOSOne_formatting_sample_main_body.pdf) **and** [**http://www.journals.plos.org/plosone/s/file?id=ba62/PLOSOne_formatting_sample_title_authors_affiliations.pdf**](http://www.journals.plos.org/plosone/s/file?id=ba62/PLOSOne_formatting_sample_title_authors_affiliations.pdf)

Reply: We have adjusted the formatting of the text, the tables, and the figures to comply with the PLOS ONE style requirements.

**2.  Please include captions for your Supporting Information files at the end of your manuscript, and update any in-text citations to match accordingly. Please see our Supporting Information guidelines for more information:** [**http://journals.plos.org/plosone/s/supporting-information**](http://journals.plos.org/plosone/s/supporting-information)**.**

Reply: We have added captions for all Supporting Information files at the end of the manuscript.

**3. Thank you for stating the following in the Competing Interests section:**

**The authors declare that the research was conducted in the absence of any commercial or financial
relationships that could be construed as a potential conflict of interest. For the purpose of this study, Freie Universität Berlin and Aurora Health signed a formal data usage agreement. The data collection was conducted with regular users of the app. As part of the data usage agreement, Aurora Health provided the authors with a raw data export file from the app’s database. Aurora Health was not involved in the preparation of the raw data, the data analysis, the writing of this article and the interpretation the results.**

**Please confirm that this does not alter your adherence to all PLOS ONE policies on sharing data and materials, by including the following statement: "****This does not alter our adherence to  PLOS ONE policies on sharing data and materials.” (as detailed online in our guide for authors** <http://journals.plos.org/plosone/s/competing-interests>**).  If there are restrictions on sharing of data and/or materials, please state these. Please note that we cannot proceed with consideration of your article until this information has been declared.**

**Please include your updated Competing Interests statement in your cover letter; we will change the online submission form on your behalf.**

Reply: We have added the statement to the Competing Interests section and included the updated statement in our cover letter.

**While revising your submission, please upload your figure files to the Preflight Analysis and Conversion Engine (PACE) digital diagnostic tool, https://pacev2.apexcovantage.com/. PACE helps ensure that figures meet PLOS requirements. To use PACE, you must first register as a user. Registration is free. Then, login and navigate to the UPLOAD tab, where you will find detailed instructions on how to use the tool. If you encounter any issues or have any questions when using PACE, please email us at figures@plos.org. Please note that Supporting Information files do not need this step.**

Reply: We have uploaded all figures to PACE and have added the adjusted files downloaded from PACE to this revision of our manuscript.

**Reviewer's Responses to Questions**

**1. Is the manuscript technically sound, and do the data support the conclusions?**

**Reviewer #1: Yes**

**Reviewer #2: Partly**

**2. Has the statistical analysis been performed appropriately and rigorously?**

**Reviewer #1: Yes**

**Reviewer #2: I Don't Know**

**3. Have the authors made all data underlying the findings in their manuscript fully available?**

**Reviewer #1: Yes**

**Reviewer #2: No**

Reply: We would like to point out that we have made all data underlying the findings fully available in the Zenodo database (DOI: 10.5281/zenodo.3384860) at https://zenodo.org/record/3384860.

**4. Is the manuscript presented in an intelligible fashion and written in standard English?**

**Reviewer #1: Yes**

**Reviewer #2: Yes**

**5. Review Comments to the Author:**

**Response to Reviewer 1**

**This paper represents a pilot study in validating the mobile app for depression screening. For an initial pilot study, it was well-designed and used appropriate statistics. The authors were appropriately careful in the Discussion to point out the pilot nature and related limitations and future directions needed. The writing was clear and the logic was easy to follow. I have just a couple of suggestions for minor additions:**

Reply: Thank you very much for your kind feedback.

**1) In terms of the Moodpath depression score, the authors acknowledge that they just chose a cutoff of 2 or higher on severity without empirical testing of whether that threshold was ideal. However, for the next criteria of the sum of all symptoms that had this “clinical significance on more than half of the ambulatory assessments” – the authors do not clarify how that criteria was chosen. Was that also just chosen without empirically determining whether ideal. For both, authors should clarify if these decisions for cut points were made a priori to inspection of the data.**

Reply: Thank you very much for pointing this out. The criterion was chosen based on the PHQ-9 algorithm method. We added the following passage to line 283:

“This rule is based on the PHQ-9 algorithm method in which only items rated as at least 2 (more than half the days) are counted towards the symptom score (68). In accordance with the PHQ-9 algorithm method, an exception was implemented for item 8 (ideas of self-harm or suicide). This item is counted if it occurred with clinical significance on at least half of the ambulatory assessments.”

We also added a sentence on another exception that was made for the symptom “ideas of self-harm or suicide” regarding the following rule to line 264: “To generate the 14-day report, a minimum of 4 assessment points for each depression symptom (Table 1) is required. This applies for all symptoms except for item 8 (ideas of self-harm or suicide) that has a minimum requirement of two assessments. The exception was introduced based on user feedback indicating that this question was too common.”

**2) Toward end of Discussion, the authors suggest that a structured clinical interview would be the ultimate criterion to use for future studies. I suggest they expand on that and note that longitudinal research is then needed to examine the predictive validity of important clinical outcomes such as response to treatment, relapse rate, subjective quality of life, quality of daily functioning, suicidal thoughts/attempts, etc. Ultimately, it would be the ability of the screening instrument to detect future behavior that would determine if it is "better" than a self-report measure that relies on retrospective recall. I agree that a first step for future research is to match to SCID diagnosis, but some discussion of further validation and potential use to predict best type of treatment and track changes during treatment would be useful too.**

Reply: Thank you for this valuable feedback. We added these thoughts to line 759 to underline that we as well see a certain potential of the approach in the field of digital phenotyping and precision mental healthcare:

“Furthermore, multimodal smartphone-based assessments may enable the identification of digital phenotypes, i.e. groups of people with distinguishable patterns of behavior assessed through smartphones sensors and interactions with the device [92]. In longitudinal study designs, digital phenotypes can be investigated in terms of their predictive validity with regard to clinical outcomes such as treatment response, treatment dropout, relapse rate, suicidality, quality of life or daily functioning. Future applications could be data-driven therapy-assistance systems that support decision making and enable precision mental healthcare [93].”

**Response to Reviewer 2**

**I have provided my detatiled comments in the uploaded document. In summary:**

**This paper reports on a diagnostic accuracy study which evaluates the ability of self‐reported ambulatory assessment of depressive symptoms and mood monitoring to correctly classify participants as experiencing Major Depressive Disorder, using the PHQ‐9 as the gold standard.**

**This paper is timely as the use of ambulatory assessment and momentary monitoring of mood symptoms is gaining in use ahead of the research evidence which should underpin it.**

**The paper is fairly well written but it is long and could be shortened to improve clarity. The main concern I have is whether there might be two papers presented as one here. Simply put, one is whether MOODPATH diagnosis agrees with a PHQ‐9 diagnosis and the other is whether mood monitoring agrees with the PHQ‐9 scores? It took me a number of times reading through the manuscript to understand exactly what was being presented and whether they actually belonged together? I just wonder if you are trying to achieve too much for one paper and clarity could be improved if you dealt with them separately? Another concern is the large number of models presented and the lack of a pre‐published protocol. This needs to be addressed.**

Reply: Thank you very much for the immensely helpful remarks. Please find our detailed replies below.

**TITLE OR ABSTRACT**

1. **The title could be altered to clearly identify this paper as a study of diagnostic accuracy. E. The diagnostic accuracy of 14‐day smartphone ambulatory assessment of depression symptoms and mood dynamics comparison with the PHQ‐9 depression screening in a population sample.**

Reply: Thank you very much for this suggestion! We are a bit hesitant to label this a study on diagnostic accuracy due to the lack of a clinical interview as a criterion. We don’t want to give the wrong impression that the app is a diagnostic instrument. Our suggestion would be to change the title to “Screening accuracy of a 14-day smartphone ambulatory assessment of depression symptoms and mood dynamics in a general population sample: comparison with the PHQ-9 depression screening”.

**ABSTRACT**

2. **The abstract overstates the background literature and could benefit from a more objective stance. Detection of cases in primary care is a complex issue. The seminal study by Thompson et al published in the Lancet in the early 2000 showed that the majority of the ‘missed cases’ of depression were at the milder end and close to diagnostic thresholds. The lack of a clear diagnostic test for depression/clear gold standard makes working out who is right and who is wrong a difficult endeavor. There is a tendency in the literature to assume that the primary care physician is ‘wrong’. I caution the authors from falling into this trap. Reconsider the use of “but cases of depression are often not detected by primary care providers” What evidence do you have for the statements: “there is a high demand for low‐threshold but clinically sound approaches to depression detection’ ? and “Recent studies show a great willingness among users of mobile health apps..”**

Reply: We agree that it is not helpful to overstate the role of specific issues within the complex set of potential barriers that may lead to undetected cases of depression. The statement “but cases of depression are often not detected by primary care providers” is based on a study by Wittchen et al. (2002) that was conducted in Germany within 412 primary care settings (15.081 patients). Here as well, the detection of mild to moderate cases was much better than for the milder spectrum. We changed the abstract accordingly. It now says:

“Major depression affects over 300 million people worldwide, but cases are often detected late or remain undetected. This increases the risk of symptom deterioration and chronification.”

Furthermore, we slightly modified the following sentence in the introduction (line 58) to indicate that especially milder cases are often missed by primary care providers:

“In addition, especially milder cases of major depression often remain undetected by primary care providers—the main and often only access point to care for the majority of patients with symptoms of depression (4).”

The statement “there is a high demand for low‐threshold but clinically sound approaches to depression detection” is based on literature, e.g. by Halfin, 2007, that points out that the risk of future episodes of depression increases with each untreated or incompletely treated past episode.

The statement “Recent studies show a great willingness among users of mobile health apps…” is based on literature, e.g. by Rubanovich et al. (2017), Torous et al. (2014) and BinDhim et al. (2015). The literature on this is summarized in the second paragraph of the introduction.

**INTRODUCTION**

3. **Scientific and clinical background could be edited to be more balanced about the state of the evidence. There is a weighting towards assuming that the approach will be beneficial (see examples below) and only a few lines ( 170‐172) (100‐107) about the potential for harm. The introduction should also include more explicitly the intended use and clinical role of the approach, being clear about the setting/population they are designing the test for. Examples in the Introduction that are overstating the evidence:**

**In Line 94: references line 734‐736 ref 19 Rubanovich to make the statement that mApps are widely used. When I checked this reference I found that Rubanovich and colleagues recruited for one year (2015‐2016) using a variety of sources (health care orgs, web ads, news stories, public transport Ads, and word of mouth, Google play store, research registries, social media, fliers and other sources. They also paid people $USD20 for completing the surveys. Despite this they report only 177 participants. The authors themselves make the statement that 300 million people worldwide experience depression. One could expect that with the huge target population and the very comprehensive recruitment strategies that Rubanovich employed they would have attracted many more participants than 177 if mApps are widely used? Unfortunately, Rubanovich does not indicate anywhere in their paper that I could see how many clicks or the possible audience they were ‘seen by’ to determine their true response rate. There is a growing tendency within the mHealth research field to overstate the use of m technologies, and it will be much more helpful for the research community if a more objective stance is taken. The potential of mhealth to make an impact is certainly there, but we are far from that reality at present and we need to build the evidence base to understand why the uptake is studies such as Rubanovich is so low.**

**Line 95 quotes Torous paper about smartphone ownership to support the statement that there is a high willingness to complete smartphone symptom screenings. When I checked this reference I found that 50% of 100 smart phone owners attending a psychiatric outpatient clinic of a large teaching hospital expressed interest in using smart phone symptom tracking. They also state that only 10% of patients took part in the survey.**

**I have not checked all the references, but the tendency to overstate the findings detracts from the importance of the current paper. I suggest that the authors more accurately report the state of the evidence.**

Reply: Thank you very much for pointing this out. It was not our intention to present a one-sided account of the current evidence.

We added the following sentences to line 92 to present a few additional issues that we consider critical when it comes to app-based mental health and especially findings of a high willingness to try such approaches:

“It is important to note that the willingness to use smartphone-based mental health services does not necessarily translate into actual use of such apps and that potential users often report concerns regarding the trustworthiness of apps in terms of their clinical benefit and privacy protection [23]. Furthermore, it becomes increasingly evident that mental health apps struggle with user engagement and retention [33]. These issues require further research on user-centred approaches to app development by focussing on the personal and contextual factors that shape usability requirements [34].”

We still think that there is sufficient evidence for a high willingness to use mental health apps in the literature. But – as stated above – this does not necessarily reflect in real world usage of such services.

Regarding the Rubanovich paper: We think it is important that participants in this study were not recruited for the survey alone but for participation in a clinical trial. This may explain the relatively long recruitment period and low number of participants.

Regarding the Torous paper: In the abstract the authors state that more than 50% of the participants in all age groups indicated interest in tracking symptoms. This is a bit misleading because figure 1 of the paper shows that the willingness was actually slightly below 70% on average and 80% in the age group 30-45. The exception here was the age group 60+. Therefore, we still think the paper illustrates that there is a high willingness to use smartphone-based symptom tracking. To further support this point, we added a more recent publication by Lipschitz et al. (2019) that found similar levels of interest.

**Line119: it would be helpful if the strengths and weaknesses of this approach could be clearly articulated here and referenced appropriately.**

Reply: We added the following paragraph to line 114 to summarize the most important strengths and weaknesses of AA:

“As summarized by Trull and Ebner-Priemer [41], distinct advantages of AA are the ability to gather longitudinal data with high ecological validity as well as the ability to investigate dynamic processes through short assessment intervals that would be difficult to assess retrospectively. Common limitations are the technical requirements, compliance with AA study protocols – e.g. due to repetitiveness, long assessments, or frequent reminders – as well as privacy concerns [41].”

4. **Study objectives and hypotheses: The two aims are clearly stated and the use of 14‐day monitoring compared with the retrospective 14 day PHQ is a strength.**

**METHODS**

**Some of the details here should be in the results e.g. N=200, lines 202‐214 are results. Reorganise the paper to be in line with the STAR D guidance. 202‐214 move to results**

Reply: We moved the description of the sample to the results section.

5. **It appears that this is a retrospective design – this is worth stating and including in the abstract. Can you explain why you decided to use MOODPATH users for this study. Can you confirm whether any of the authors have any connection to Moodpath or Aurora**

Reply: We do not think it is a retrospective design because the PHQ-9 was assessed after the 14-day screening, specifically for the purpose of this study. Moodpath was chosen because it was at the time the largest and fastest growing German language depression screening app on the app store. Furthermore, Aurora agreed to a formal data sharing agreement with Freie Universität Berlin that provided us with full autonomy in the analyses and publication of the findings.

Aurora is one of several Berlin based start-up companies that collaborate with Freie Universität Berlin to conduct practice-oriented research on e-mental health solutions that were developed for large scale implementation. We believe that it would severely limit the validity of research on e-mental health and especially on mobile mental health if apps by commercial companies were to be ignored by researchers. Such collaborations are always based on legal agreements that ensure our full independence as researchers. As stated in the declaration of interests, the research was conducted in the absence of any commercial or financial relationships that could be construed as a potential conflict of interest.

**PARTICIPANTS**

**Please organise the participants section in line with the STARD flow as below.**

Reply: We have re-organised the section accordingly.

6. **Eligibility criteria – users of Moodpath App and agree to consent – can this be stated clearly?**

Reply: We adjusted the text to state this more clearly in line 194.

7. **On what basis potentially eligible participants were identified. This is well described.**
8. **Where and when potentially eligible participants were identified (setting, location and dates)**

Reply: We moved the following sentence to comply with the STARD flow to line 199: “All users who completed the app’s regular 14-day screening phase were contacted automatically through an in-app notification until the sample size of 200 was reached.”. And we added the following sentence to line 201 to clarify the recruitment date: “The recruitment took place in January 2017.”.

9. **Whether participants formed a consecutive, random or convenience series. Please make it clear that this is a convenience sample. Please make it clear that this is a convenience sample.**

Reply: We added this to line 190.

**TEST METHODS**

10. **Index test, in sufficient detail to allow replication**

**You have two index tests MOODPATH and the mood dynamics – or is the mood dynamics a part of the MOODPATH assessment.**

Reply: The app contains two components, 1) the depression screening and 2) the mood tracking. The depression screening was the index test in this paper. The mood tracking was secondary and investigated in exploratory analyses.

**In Table 1 are they Yes, No answers? Can you include this in the table?**

Reply: As stated in the text, “Participants answer each question, e.g. “Are you feeling depressed?”, on a dichotomous scale with “Yes” or “No”. If a symptom is confirmed, the severity (“How much does this affect or burden you?”) is rated on a four-point scale ranging from 1 to 4 with a visual anchor (see figure 1).”. We added this information to table 1.

**Line 224 – provide reference for usability testing results**

Reply: The usability testing results are not published. We removed the sentence.

**Line 226 – provide reference for Aurora**

Reply: Since the Aurora platform is not the subject of the paper and since there is no published research on the system, we have removed the sentence.

**Line 229 – provide the 45 questions, even if as a supporting information document – readers need enough information to replicate your study.**

Reply: We added a list of all 45 questions used in the study as supporting information (see S6 Table).

**Line 240 – can you explain how the 3 questions are selected? Really non‐randomly? Do people cover all 45 questions over the 14 days in equal amounts? I found this difficult to follow. Can this be explained more clearly? Line 256‐258 explains this more clearly, but this whole section could benefit from clarity and perhaps a diagram to allow replication.**

Reply: Thank you for pointing this out. We did not provide enough detail to replicate the exact procedure used to select the question blocks. In the revised manuscript, we added the following explanation to line 248:

“The order and content of the question blocks was predefined and identical for each participant (S7 Table). Blocks were divided into obligatory blocks and optional blocks. All obligatory blocks combined contained the minimum of questions required to generate the 14-day report (see below). If an obligatory block was missed, it automatically took the place of the same block on the next day (e.g. next day’s morning block if a morning block was missed).”

Furthermore, we adjusted the following sentence in line 270:

“In order to still ensure enough data points for each symptom, the differentiation into obligatory and optional blocks was introduced. This system ensured that missed blocks containing questions on depression symptoms for which a minimum of 4 assessments was not reached yet were repeated.”

Finally, we added the complete schedule for the blocks used in this study as supplementary material (S7 Table). In combination with the complete list of questionnaires, the reader now is provided with all information required to replicate the study.

**Line 265 – explain how you ascertained ‘clinical significance’ for each item**

**Please justify and provide a reference to support how MOODPATH came up with all the rules they have for allocating a score?**

Reply: Since this is the first study on this approach, there was no evidence to base this criterion on. A severity score of 2 or higher was chosen because it appeared as neither too strict nor too lenient. Unfortunately, the study was not pre-registered at the time. We added the following to the limitations section to further underline this issue:

“Thirdly, since this was the first study on a new approach to depression screening there were no pre-existing findings to base cut-off criteria on. At this stage, the findings must be seen as preliminary and require replication – ideally in pre-registered trials.”

**Line 280 – provide justification and a reference for this approach**

Reply: Since this is the first study on the Moodpath depression screening, the criterion of 2 core + 3 associated symptoms was not based on previous literature on this particular screening approach. However, it was decided to set the cut off at 5 symptoms based on the PHQ-9 algorithm method described in line 318.

**Line 285‐296 –The PHQ‐9 is a well validated scale but I found this section a little confusing. The original paper is the Spitzer, JAMA 1999 paper which outlines the process for the original algorithm scoring for Major Depressive Disorder – which I noted to related to DSM IV criteria rather than DSM5?.**

Reply: It is true that the cited papers refer to the DSM-IV. However, to our knowledge, the PHQ-9 did not receive a revision when the DSM-5 was released because there were (with one exception) no changes for the depression diagnosis. In particular, the list of potential symptoms used to diagnose depression – which the PHQ-9 is based on – were not changed. To be 100% precise, we adjusted the following sentence in line 315:

“In addition, an algorithm can be used to approximate a provisional diagnosis on the basis of the DSM-IV diagnostic criteria (70) – which are (with the exception of an adjustment to the bereavement exclusion criterion) identical to the DSM-5 criteria.”.

**My reading of the Ref no 69 &70, is that that they provide support for using the cut‐off point of >=10; but they do not appear to be an appropriate reference for using the Algorithm scoring.**

Reply: Thank you very much for pointing this out. We intended to cite Manea et al. (2015) here but cited a different paper by the same authors by accident. The reference was corrected in the revised manuscript.

**Can you revise this section and provide details as to whether you used the PHQ 9 as shown in the Appendix of Kroenke article Ref 32? Did you administer the section about “… checking off how difficult these problems made it to do your work, take care of things at home etc” This was not clear to me. How did you apply the algorithm? Please include the exact question list used as the PHQ 9 and the rating scales so replication is possible.**

Reply: We revised this section to make it clearer how we assessed the PHQ-9 (see line 309):

“For the initial validation of the Moodpath screening, the established PHQ-9 questionnaire with 9 questions as published by Kroenke et al. (32) was chosen in its German translation (69) as reference criterion due to strong evidence on its screening utility (70, 71). The additional PHQ-9 question on functioning (32) was not asked.”

Since the questionnaire is freely available and the German translation used in this study is cited in the manuscript, we did not add it as an appendix.

11. **Rationale for choosing the reference standard (if alternatives exist). This is explained, but you could provide a little more commentary on the reasons and perhaps the alternatives. I was also confused as to why, considering you use Ref 69&70, that you did not decide to use the cut‐off of 10?**

Reply: As stated in the paper, the reason to pick the algorithm method was to approximate a provisional diagnosis based on the DSM-5/DSM-IV criteria. The incorrect reference 70 was replaced with a paper on the algorithm method applied in this study.

**12a Definition of and rationale for test positivity cut‐offs or result categories of the index test, distinguishing pre‐specified from exploratory**

**Can you explain what you had pre‐specified and what was exploratory? I find lines 300‐315 hard to follow. Please make is very clear which PHQ9 scoring method you have used and which cut‐off you are using.**

Reply: In this study, we looked at two approaches to calculating a Moodpath cut-off. The first approach was to use the ICD-10 criteria for moderate depression, taking into account core and associated symptoms. The second approach was exploratory in nature and aimed to identify the ideal Moodpath depression score (i.e. the number of symptoms that fulfilled the criteria for clinical significance in this trial) without taking into account core and associated symptoms. We adjusted the following sentence in line 335 to make this clearer:

“In addition, exploratory methods were applied to identify the ideal Moodpath sum score cut-off without taking into account ICD-10 criteria for core and associated symptoms. To this end, sensitivity and 1-specificity for different Moodpath depression scores were plotted as a receiver operating characteristic (ROC) curve, which provides the area under the curve (AUC) as a measure of accuracy.”

**Line 305: was the cut‐off of 5 symptoms pre‐specified? If not, please explain whether you did prespecify a sample size and potential cut‐offs that might be clinically meaningful. Explain how you decided these.**

Reply: The PHQ-9 algorithm method cut-off was pre-specified. It is based on the Manea et al. (2015) paper that was accidentally cited incorrectly. We added the reference here again to underline that the approach is based on that paper.

**I became confused when I read Line 560 “The ICD‐10 cut off score of 5 symptoms was used….” Can you clarify?**

Reply: In line 560 we refer to the cut-off for the Moodpath depression score not to the cut-off for the PHQ-9.

**Line 308‐ how did you choose the MOODPATH cut off and can state more clearly what it is?**

Reply: The Moodpath cut-off was set to 5 based on the ICD-10 criteria for moderate depression, taking into account the requirement for 2 core + 3 associated symptoms. This is stated in line 291 of the manuscript:

„Users of the app receive a report on the severity of their symptoms that takes into account the Moodpath depression score as well as the ICD-10 criteria for depression (67): i.e. 2 out of 3 core symptoms + 2 out of 7 associated symptoms were reported as indication of “mild depression”, 2 core + 3 or 4 associated symptoms were reported as “moderate depression” and 3 core + 5 or more associated symptoms were reported as potential “severe depression”. With the report, users received the following disclaimer: “The Moodpath App screening is not a substitute for a medical diagnosis. It should only be used as an indication to seek professional consultation”. For the purpose of validation, in this study the above described ICD-10 criteria for “moderate depression” (i.e. 2 + 3) were used as indication of clinically relevant symptoms.“

**12b Definition of and rationale for test positivity cut‐offs or result categories of the reference standard, distinguishing pre‐specified from exploratory**

**Can you clearly state the definition and rationale for any pre‐specified PHQ‐9 Cut‐offs you planned to use?**

Reply: As stated in line 331: “The specificity of the Moodpath screening was calculated as the percentage of participants correctly classified as not depressed out of all participants with a PHQ-9 result below the clinical cut-off.”.

This was the only PHQ-9 cut-off we planned to use in this study.

**13a Were clinical information and reference standard results available to the readers of the MOODPATH results?**

Reply: The 14-day report summarized the results by presenting core and associated symptoms as defined in the ICD-10. The report further explained that the result was not a clinical diagnosis and suggested next steps (e.g. seeking mental health care) based on recommendations by the German Society for Psychiatry and Psychotherapy, Psychosomatics and Neurology (DGPPN) that are written by experts in the field and are published as official guidelines (“Nationale Versorgungsleitlinie für unipolare Depression”).

**13b Were clinical information and index test results were available to the assessors of the PHQ‐9 results?**

Reply: The PHQ-9 was assessed online. No assessors were involved.

**Did you write a separate detailed analysis plan before the analysis was conducted? Please provide this as a supplementary file.**

Reply: No, we didn’t write a separate analysis plan before the analysis.

**ANALYSIS**

**14 Methods for estimating or comparing measures of diagnostic accuracy – these are explained.**

**Line 340: Can you justify the decision to calculate 14 separate regression models? How did you account for the use of multiple models? Ensure you discuss this adequately in the discussion.**

Reply: As stated in the methods section, these analyses were exploratory in nature. The reason was that (see line 375): “This allowed further explorative analyses on the influence of the duration of ambulatory data collection on the predictive validity of the calculated indicators of mood dynamics.”

We added the information that the analyses were exploratory in line 647 in the discussion to ensure that the reader is aware that the findings are not intended to test hypotheses. To account for multiple models, we adjusted the significance level to p<.01.

**15 Explain how indeterminate MOODPATH and PHQ9 results were handled. It would help to include a sub‐heading about this and about missing data so the reader can easily find this detail.**

Reply: Due to the clearly defined cutoffs, there were no indeterminate results. The handling of missing data for the Moodpath screening is closely linked to the question block schedule (i.e. ensuring that missing obligatory blocks were repeated). Therefore, we decided to explain the procedure in Moodpath depression screening section instead of under a separate sub-heading. There was no missing data on the PHQ-9.

**16 Explain how missing data on the index test and reference standard were handled**

Reply: Please see our reply to point 15.

**17 Any analyses of variability in diagnostic accuracy, distinguishing pre‐specified from exploratory**

**Line 303: the use of the term “ICD‐based MOODPATH screening criterion of 2 core + 3 associated symptoms” is cumbersome and I found this section a little confusing. It could be written more clearly so the reader is very clear about what you are comparing with what and just how many analyses are being conducted. The use of sub‐headings, tables etc should be considered to increase the clarity of this section. Be consistent with terminology and consider a table of definitions to help the reader.**

Reply: We are sorry that this section was confusing. The Moodpath Depression screening data was handled in two ways:

Approach 1: The symptoms were grouped into core and associated symptoms as defined in the ICD-10. For each participant it was checked whether at least 2 core and 3 associated symptoms were given above the threshold. This was then used to calculate sensitivity and specificity using the PHQ-9 based criterion described in line 318. This is explained in the first paragraph of the “Psychometric evaluation of the depression screening” section.

Approach 2: A sum score of all symptoms was calculated, without differentiating between core and associated symptoms. This was then used for the ROC curve. This is explained in the second paragraph of the “Psychometric evaluation of the depression screening” section. To make it clearer, we adjusted the following sentence in line 335:

“In addition, exploratory methods were applied to identify the ideal Moodpath sum score cut-off without taking into account ICD-10 criteria for core and associated symptoms.”

**18 Intended sample size and how it was determined ‐ Please include details and justify your decision**

Reply: We added the following sentence to line 191:

“The sample size was chosen based on the estimated number of app users to complete the 14-day assessment within a period of approximately one month.“

**RESULTS**

**19 Please include a diagram that shows the flow of participants.**

**Include information about the number of people approached.**

**Account for all participants from the 200 to the study sample and through the data waves.**

**Provide information on the MOODPATH participants – is the 113 used here representative? Please move the results about the MOODPATH participants to results: In particular move lines 202‐214 to results.**

Reply: Thank you for this suggestion. We have added Fig 2 with the participant flow diagram.

The number of participants approached was 200. As stated in line 199: “All users who completed the app’s regular 14-day screening phase were contacted automatically through an in-app notification until the sample size of 200 was reached.”

We moved lines 202-214 to the results section (see line 385). All available information on the participants are presented in the paper.

**20 Baseline demographic and clinical characteristics of participants**

**‐ Please include**

Reply: Demographics are presented in line 395.

**21a Distribution of severity of disease in those with the target condition**

**‐ Please include baseline data**

Reply: This is presented in the “Descriptive statistics” section (line 401)

**21b Distribution of alternative diagnoses in those without the target condition**

**‐ Please provide any details that you have about these, especially noting that the use of PHQ‐9 pre‐supposes that there is no physical reason for the symptoms reports.**

Reply: Unfortunately, we don’t have any additional clinical data on the study participants.

We have added this as a limitation by adding to the following sentence in line 572:

“The sample was not recruited to be a clinical sample and there is no information on participants’ treatment or depression history or on potential physical reasons for reported symptoms.”

**22 Time interval and any clinical interventions between index test and reference standard**

**‐ Please provide details**

Reply: Unfortunately, we do not have any details on clinical interventions that took place during the 14-day assessment phase. This is included in the limitations section.

**This paper is presenting the comparison of MOODPATH to PHQ9 and MOOD tracking. Would it be preferable to separate out these two into separate papers?**

Reply: We think that both parts of the study should be presented in combination because the combination of retrospective self-report with ambulatory assessment constitutes the unique possibilities of an integrated, smartphone-based approach to depression screening.

**23 Cross tabulation of the index test results (or their distribution) by the results of the reference standard**

**‐ This appears in Table 2 at line 994. Please label Table 2 more clearly so it is in line with STARD guidance.**

Reply: We splitted Table 2 and changed the title of the new Table 3 to “Cross tabulation of the index test results.”

**24 Estimates of diagnostic accuracy and their precision (such as 95% confidence intervals)**

**This is included as Table 4. Please include diagnostic accuracy in the title to assist the reader to find this information easily, in line with STAR D recommendations**

Reply: As with the title of the study, we wouldn’t want to imply that the app was a diagnostic instrument.

**25 Any adverse events from performing the index test or the reference standard**

**‐ Please include information about how you assessed this.**

**‐ Include any information you have about about drop‐outs**

Reply: We did not assess adverse events and we did not follow-up with participants who decided not to participate in the study.

**DISCUSSION**

**26. The discussion would benefit from being shortened considerably.**

**There should be a section on the conundrum faced in mental health – that there is no objective gold standard, and even the best is subject to bias. This complicates all such studies.**

Reply: We think that this issue is already sufficiently addressed in the limitations section. This would however be an important point to discuss in a future paper that compares the Moodpath screening with a clinical interview.

**27. MOODPATH – has this been developed using a rigorous process? Comment on the strengths and weaknesses of this assessment tool. Is there more work to be done?**

Reply: As stated in line 595 “(…) apps like Moodpath are constantly being improved based on research results and user feedback. Consequently, the screening algorithm and other elements of the app are likely to change in the future. Therefore, future versions of the app may work differently than the version 2.0.7 described in this paper.”

In the discussion, we address the – in our opinion – key strength (i.e. the promising screening performance) as well as weaknesses (e.g. a negative trend in mood ratings over time that could be an indicator of a burden of self-tracking or the lack of validated cut-offs).

We also discuss the future work to be done in the “Conclusions” section.

**Please comment on the study sample and how generalisble the findings are.**

Reply: As stated in the limitations section (line 585):

“Fifthly, the study data were collected in a self-selected anonymous sample. The sample was not recruited to be a clinical sample and there is no information on participants’ treatment or depression history or on potential physical reasons for reported symptoms. Different recruitment methods can result in systematic differences between samples of participants with depression symptoms [80]. Even though the PHQ-9 scores indicated a high symptom load, the sample needs to be treated as a non-representative population sample and furthermore needs to be contrasted with samples recruited in clinical settings in the future. Consequently, the generalizability of the results is limited. Furthermore, demographic data indicates that the sample was relatively young and mostly female, which further reduces generalizability to other demographic groups”

**Comment on regression to the mean.**

We are not sure what exactly this refers to and would need a more detailed explanation to be able to reply to this point.

**Please comment on the multiple regression models and the problems with multiple testing – how has this influenced your findings?**

The multiple testing did not influence the findings because all results were statistically significant at the p <.01 significance level. We added the following sentence to line 379:

“The main research interest behind these exploratory analyses was to obtain information on how the duration of the ambulatory assessment affects the predictive validity of the data. To account for the problem of multiple testing, we adjusted the significance level to p<.01.”

**OTHER INFORMATION**

**28. Registration number and name of registry**

**‐ Please provide**

Reply: The study was not pre-registered.

**29. Where the full study protocol can be accessed**

**‐ I could not find any information about the study protocol**

Reply: No study protocol was published for this study.

**30. Sources of funding and other support; role of funders**

**Can you confirm whether any of the authors have any connection to Moodpath or Aurora?**

Reply: This is fully disclosed in the conflict of interest statement:

“The authors declare that the research was conducted in the absence of any commercial or financial relationships that could be construed as a potential conflict of interest. For the purpose of this study, Freie Universität Berlin and Aurora Health signed a formal data usage agreement. The data collection was conducted with regular users of the app. As part of the data usage agreement, Aurora Health provided the authors with a raw data export file from the app’s database. This does not alter our adherence to PLOS ONE policies on sharing data and materials. Aurora Health was not involved in the preparation of the raw data, the data analysis, the writing of this article and the interpretation the results.”
