# Supplementary material for: Screening accuracy of a 14-day smartphone ambulatory assessment of depression symptoms and mood dynamics in a general population sample: Comparison with the PHQ-9 depression screening
Source: PLoS One. 2021 Jan 6;16(1):e0244955. doi: 10.1371/journal.pone.0244955 (PMC7787464; doi:10.1371/journal.pone.0244955)
Supplement: Attachment — Submitted filename: Response_to_reviewers_2020-12-18_SB.docx [file pone.0244955.s010.docx]

**Response to editor**

**Additional Editor Comments:**

The reviewers indicated that all former comments have been addressed. However, a new reviewer suggested some minor revisions to further improve the manuscript, and I particularly agree on the need of more clarity about the recruitment process and that a Flow-chart would help to address this issue.

Thank you very much! Please find our replies to reviewer 3 below.

We also used this opportunity to add the following paragraph in the discussion on the latest findings regarding the limited incremental value of affective dynamics in research on personality, well-being and psychopathology (see line 675):

“Still, it is important to note that the incremental value of affective dynamics in research on personality, well-being and psychopathology is increasingly being questioned by studies that aggregate datasets on indicators of affect dynamics [86-89]. In line with our findings, these studies identify mean level and general variability as the main indicators but find only little additional variance explained by other indicators of affect dynamics.”

**Response to reviewers**

**Reviewers' comments:**

**Reviewer #3:** This is an important, novel paper which goes some way to addressing the kinds of questions associated with the surfeit of mobile apps available to people with mental health difficulties and their clinicians. The paper has described clearly and accurately the literature and does a mostly thorough job in doing so.

The study itself appears to be appropriate in terms of design and statistical analysis and the conclusions are balanced and realistic. I believe that the previous reviews have been addressed satisfactorily. I did feel there were some issues around the clarity of the methodology some of which could probably be addressed with figures rather than text.

A few comments:

1) Line 75: "The willingness to seek mental health information online was found to be higher among persons who experience increased psychological distress" - Higher compared to whom?

Thank you for pointing out this missing piece of information. We adjusted the sentence accordingly:

“Among internet users in a random sample of general practice patients in Oxfordshire (England), the willingness to seek mental health information online was found to be higher among persons who experience increased psychological distress and even higher in persons with a past history of mental health problems [17].”

2) Line 85 - poorly constructed sentence. Remove 'for' and consider changing the word obscure for something other such as opaque, complex or hard to navigate

We agree that the wording in this sentence is not ideal and have adjusted the sentence as follows:

“However, the number of freely available mental health apps has long become opaque for clinicians and end users seeking trustworthy, secure and evidence-based apps [21, 27, 28].”

3) Page5 - What are the figures for health or other app use in the general population - how does this compare to mental health app use?

To our knowledge, there are no population-based studies on mental health app use in the general population of a country. However, it can be assumed that – compared to the widespread use of smartphones (up to 90% of the population, depending on the country) – mental health app use is still relatively rare. In a German population-based study (Ernsting et al., 2017), 20,54% of smartphone owners had used health related apps (most commonly for smoking cessation, weight loss or a healthy diet). Mental health apps were not covered separately, but Baumel et al. (2019) showed that there is currently only a very small number of mental health related apps that achieved more than 100.000 total downloads (worldwide). In addition, the authors showed that most of these apps struggle with very low adherence rates. At the same time, mental health apps seem to become more relevant for those who experience psychological distress or who are already suffering from a psychological disorder. To keep the introduction concise, we included findings on this (see page 4) but did not go into more detail on the use of (health) apps in the general population.

4) Page 8 - summary of pilot study aims could be more clear for the reader - strip back to the basics here.

We shortened this section and reduced the level of detail to make it easier for the reader to identify the main aims of the study.

5) Page 9 - more clarity needed over recruitment and when and how were onboarded - a diagram here would be helpful to explain the process. For example its not immediately apparent when participants start the study. If they are already users - does this impact their results and feedback? Do new users show greater effect from a usage and depression point of view?

We agree that this requires more clarity because it is very relevant to understand that the participants in our study were already regular users of the app before they were asked to participate in the study. This applies to all participants in the study. Therefore, we added the following explanation on page 9:

“Participants in this study were regular users of the app who had started using the app before being asked to participate in the study.”

Consequently, the in-app data was initially not gathered in a study context but in a regular app use context and participants retrospectively provided consent for using this data in the study. To further illustrate this, we have expanded Fig 2 by adding information explaining that – prior to the study – participants were regular users who completed the app’s ambulatory assessment phase. These users were then contacted automatically through an in-app notification and asked to participate.

Only data of app users who provided consent to participate in this study is included in the paper. Therefore, we cannot provide descriptive statistics on the user flow prior to giving consent.

Since we do not have a comparison group, it is not possible to say whether regular and study users differ in usage or symptom patterns. All participants were regular app users before participating in the study by answering the PHQ-9 items and we therefore assume that the findings might have a higher ecological validity compared to a different study design with sample of non-app users who are invited to install the app for the study.

6) Page 10 - the app has already received a CE - does this mean the app has already undergone validation testing for safety and effectiveness?

In 2017, the manufacturer of the app received a CE medical device certificate as a Class I medical device. To our knowledge, at the time this did not require pre-existing validation or effectiveness studies on the product itself. Additional studies on the app have been and are being conducted. But to our knowledge these studies are not published yet. Our study is the first one to investigate the psychometric properties of the app’s screening approach.

7) Page 16 - Is the correction type for multiple testing enough here?

We think the approach is sufficient in this case. A total of 155 tests are being conducted as part of the explorative analysis. At the chosen p < .01 we can expect that approximately two tests are false positives.

8) Page 18 and conclusions - Respondents were more likely to complete the tracker in the evening - how did this affect mood - changes in hormone levels, diurnal variance etc. should be considered here perhaps? could this be stratified?

This could result in a bias if mood scores at the evening assessments differed significantly from mood scores at midday or evening. We tested this and did not find statistically significant differences between the three assessment periods in the data. Therefore, we did not apply measures to control for this type of bias and did not include potential explanations for daytime specific variation on mood ratings in the discussion.
